# Hormonally Silent Multifocal Pheochromocytoma in the Setting of a Well-Differentiated Neuroendocrine Tumor of the Appendix: A Case Report

**DOI:** 10.7759/cureus.59295

**Published:** 2024-04-29

**Authors:** Patrick T Weldon, Megan McNally

**Affiliations:** 1 General Surgery, University of Missouri Kansas City, Kansas City, USA; 2 Surgical Oncology, Saint Luke’s Hospital, Kansas City, USA

**Keywords:** variant of uncertain significance, appendiceal neuroendocrine tumor, nonfunctional paraganglioma, hormonally silent pheochromocytoma, multifocal pheochromocytoma

## Abstract

Pheochromocytomas (PCCs) and paragangliomas (PGLs) represent tumors arising from chromaffin cells of the adrenal medulla and extra-adrenal sympathetic paraganglia, respectively. PCCs commonly produce one or more catecholamines (epinephrine, norepinephrine, and dopamine), but rarely are they biochemically silent. PGLs on the other hand, generally do not produce catecholamines. They have the highest heritability of all adrenal tumors and are known to be associated with genetic mutations. Patients with hereditary tumors typically present at a younger age and with multifocal disease when compared to sporadic disease. Specific genetic mutations have been well established with hereditary syndromes involving PCC/PGLs. Further research has aimed to identify other mutations and delineate specific phenotypes associated with these mutations.

A 34-year-old woman presented for evaluation following a laparoscopic appendectomy that identified a 4-cm well-differentiated neuroendocrine tumor on final pathology. Further work-up included a repeat CT scan followed by a Dotatate PET CT scan which revealed a large (7.3 x 5.8 cm) periaortic mass related to the left adrenal gland. Functional adrenal work-up was negative and her Chromogranin A level was 679 ng/mL. She did report intermittent chest tightness and palpitations but was otherwise asymptomatic. The patient subsequently underwent an exploratory laparotomy with left adrenalectomy and adjacent tumor resection as well as completion of right hemicolectomy with ileocolonic anastomosis. Surgical pathology revealed two distinct masses consistent with multifocal PCC. No residual tumor was found in the colectomy specimen and 24 lymph nodes were negative. She had an uneventful recovery and genetic testing showed a variant of uncertain significance for the POLE and VHL genes. She has received genetic counseling and will be enrolled in an appropriate surveillance protocol.

## Introduction

Pheochromocytomas (PCCs) and paragangliomas (PGLs) represent tumors arising from chromaffin cells of the adrenal medulla and extra-adrenal sympathetic paraganglia, respectively. PCCs commonly produce one or more catecholamines (epinephrine, norepinephrine, and dopamine), but rarely are they biochemically silent. PGLs on the other hand generally do not produce catecholamines [1]. The clinical relevance of the overproduction of catecholamines is that long-term exposure at levels higher than normal can ultimately lead to refractory hypertension as well as negative cardiovascular impact. More importantly, when they are hormonally silent these negative effects can be occurring without evidence to the patient or practitioners. PCCs and PGLs have the highest heritability of all adrenal tumors (about 40% are due to germline mutations) [2,3]. Patients with hereditary tumors typically present at a younger age and more commonly with multifocal disease when compared to those with sporadic disease [1]. Specific genetic mutations were first identified in the late 1980s associated with hereditary syndromes including von Hippel-Lindau disease (VHL gene), multiple endocrine neoplasia type 2 (RET gene), and neurofibromatosis type 1 (NF1 gene) [4,5]. Further research has aimed to identify additional genetic mutations and delineate specific phenotypes associated with these mutations.

It is recommended that initial biochemical testing should include measurement of plasma-free metanephrines or urinary-free metanephrines with a positive result typically being more than two to three times the upper limit of normal [1,2]. Once there is clear biochemical evidence, imaging is used to localize the disease. CT with intravenous contrast is usually the initial imaging modality with a sensitivity of 88%-100% [1]. Functional imaging is an important adjunct in those suspected to have metastatic disease. Advances in imaging techniques led to the development of [68Ga]-DOTATATE PET/CT which has been shown to have a lesion-based detection rate of 98.6% and is emerging as a superior form of functional imaging [6]. All patients with PCC/PGLs should be offered genetic testing [1]. The reason is that identifying the molecular cause has implications for future tumor risk and risk of malignancy. Additionally, hereditary susceptibility can have an impact on a patient's family members. Surveillance recommendations continue to evolve as longitudinal genetic data accumulates [7]. Definitive management requires surgical resection, with recommendations for minimally invasive approaches being preferred except in the cases of large (> 6 cm) or invasive disease [1].

## Case presentation

A 34-year-old woman with a past medical history of endometriosis and Gilbert’s syndrome was incidentally identified to have an abnormal appearing appendix on diagnostic laparoscopy for evaluation and treatment of endometriosis in the setting of infertility. After identification of the abnormal appendix, appendectomy was recommended but delayed initially due to pregnancy. Ultimately, she underwent a laparoscopic appendectomy. Pathology revealed a 4-cm well-differentiated neuroendocrine tumor and one lymph node was noted to be positive.

**Figure 1:**
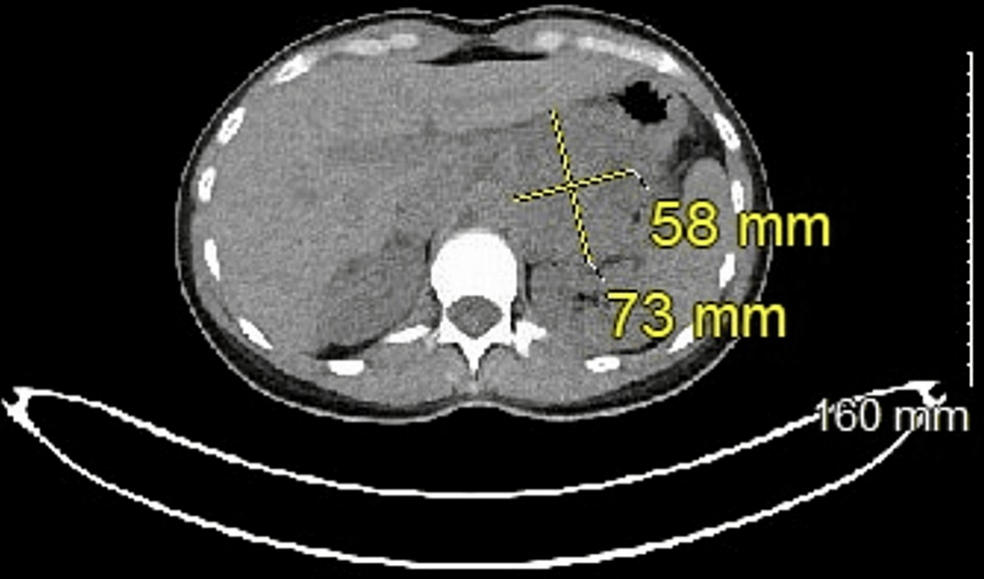
Computed tomography showing 7.3 x 5.8 cm left adrenal tumor

**Figure 2:**
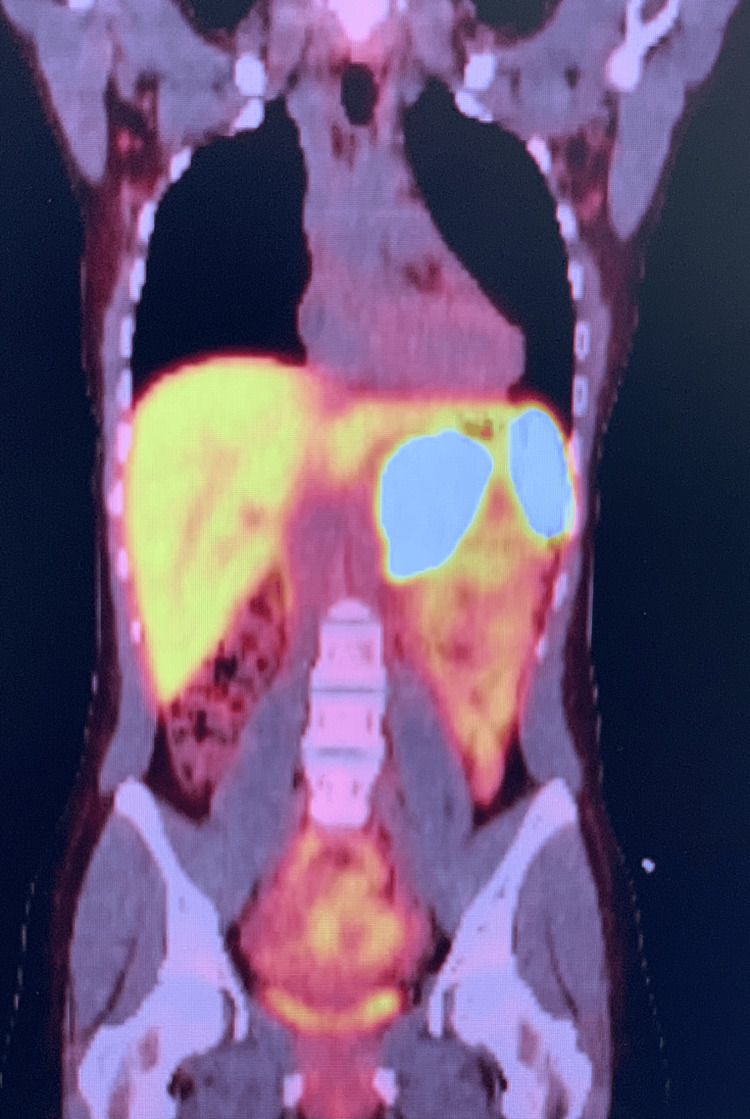
Nuclear medicine dotatate positron emission tomography-computed tomography

Following this result, she had further metastatic work-up including a repeat CT scan followed by a Dotatate PET/CT scan which revealed a large (7.3 x 5.8 cm) periaortic mass closely related to but not definitively associated with the left adrenal gland (Figures 1, 2). Functional work-up was negative including plasma free metanephrines and fractionated catecholamines. Her Chromogranin A level was 679 ng/mL. She displayed no associated carcinoid syndrome symptoms, although she did report intermittent chest tightness and palpitations.

The patient underwent an exploratory laparotomy with left adrenalectomy and adjacent tumor resection as well as completion of right hemicolectomy with ileocolonic anastomosis. Perioperative octreotide infusion was utilized prophylactically to prevent any carcinoid crisis. Of note, the left adrenal gland was in an anatomically variant location overlying the renal hilum rather than superior to the kidney. The adjacent mass was initially thought to be a lymph node with metastases. Final pathology revealed two distinct masses consistent with multifocal PCC. The dominant mass measured 7.4 cm, Ki-67 1.8%, SSTR2 positive, SDHB intact. The additional mass measured 2.8 cm. No residual tumor was identified in the colectomy specimen and 24 lymph nodes were negative. She recovered well requiring a five-day hospital stay.

Genetic testing was ordered and returned a variant of uncertain significance (VUS) for POLE and VHL genes. She is following up with her Endocrinologist who has recommended a six-month evaluation with imaging and tumor markers (both PCC and appendiceal neuroendocrine tumor) for the first two years postoperatively with consideration of decreasing towards annual follow-up for at least 10 years.

## Discussion

PCC/PGL remains a very rare disease with a wide range of clinical presentations making recognition and diagnosis challenging. Prevalence in patients with hypertension in outpatient settings varies between 0.2% and 0.6%. Autopsy studies demonstrate undiagnosed tumors in 0.05%-0.1% of patients. Approximately 5% of incidentally found adrenal masses on imaging prove to be PCC. Therefore, it is important to have a high index of suspicion to recognize and treat these tumors as exposure to hypersecretion of catecholamines can lead to increased cardiovascular morbidity and mortality. Additionally, identifying a molecular cause of these lesions can have a significant impact on family members and categorize the increased risk of other malignancies [1].

After biochemical confirmation, imaging should be performed to localize disease, with an initial CT scan of the abdomen and pelvis with intravenous contrast as well as consideration for functional imaging. [68Ga]-DOTATATE PET/CT is emerging as the superior functional imaging modality [6]. In the setting of negative biochemical work-up but imaging evidence, such as our case, it is imperative to repeat biochemical studies to ensure no laboratory/processing error. If identified to have elevated metanephrines, either selective or nonselective α blockade is utilized preoperatively to safely prepare patients for surgical resection. While there is no significant difference in morbidity or mortality between selective and nonselective α blockade, selective blockade (doxazosin, prazosin, terazosin) is associated with more intraoperative hemodynamic instability while nonselective blockade (phenoxybenzamine) results in more postoperative hypotension [2].

Genetic testing is imperative when managing these patients as up to 40% may be associated with a hereditary syndrome. The association of PCC/PGL with von Hippel-Lindau syndrome, multiple endocrine neoplasia type 2, and neurofibromatosis type 1 have been well known for some time, several other susceptibility genes have been defined in recent years [5,8,9]. The identification of mutations in genes coding for subunits of the enzyme mitochondrial succinate dehydrogenase (SDH) has led to further characterization of syndromes with defined genotype-phenotype profiles [9,10]. As our knowledge of genetics continues to improve, undoubtedly more gene mutations will be identified. Certain variants of unknown significance may ultimately end up being implicated in this disease process. Regarding the VUS in the VHL gene in our patient, specifically p.E16* c.46G>T, it is unknown if this change affects the protein’s function so the risk of malignancy cannot be established.

Evaluation and management utilizing a multidisciplinary approach are critical to the appropriate management of these patients. Whether there is confirmed genetic evidence of a mutation associated with a known PCC/PGL syndrome or not, a surveillance plan should be formulated in a patient-specific manner. Finally, patients should be sufficiently counseled on family member’s risk so they can undergo testing if indicated or desired.

## Conclusions

PCC/PGL remains a rare entity. They can be difficult to diagnose as they can have continuous hormone secretion, intermittent secretion, or more rarely be hormonally silent. Diligent work-up is needed to ensure appropriate preoperative management. Our patient’s hormonally silent multifocal PCC was identified incidentally in the workup of a neuroendocrine tumor of the appendix. Despite no clinical symptoms precautions were taken perioperatively to prevent any hormonal crisis. Genetic testing will continue to progress and provide new information to help identify those at risk as well as increase treatment options for more advanced diseases. Postoperative surveillance is warranted to ensure no recurrence and allow identification of additional tumors and should be patient specific. A multidisciplinary approach and management at higher-volume institutions can improve patient care and outcomes.
